# Financial trading decision model based on deep reinforcement learning for smart agricultural management

**DOI:** 10.7717/peerj-cs.3196

**Published:** 2025-09-12

**Authors:** Di Fan, Nazrul Hisyam Ab Razak, Wei Ni Soh

**Affiliations:** 1 School of Business and Economics, Universiti Putra Malaysia, Kuala Lumpur, Malaysia; 2 School of Management, Zhongyuan Institute of Science and Technology, Xuchang, China

**Keywords:** DQL, Trading decisions, Multifactor features, Intelligence, Sliding window

## Abstract

This study proposes a decision-making model based on deep reinforcement learning (DRL) for agricultural financial transactions, addressing core challenges such as significant data noise, strong time-series dependence, and limited strategy adaptability. We developed a multifactor dynamic denoising framework by integrating the Grubbs test for outlier detection and the median absolute deviation (MAD) method for noise handling. This framework categorizes agricultural financial indicators into six feature types, significantly enhancing robustness against data noise and improving model reliability. Furthermore, an long short-term memory (LSTM)-enhanced DRL architecture is employed, incorporating a sliding window mechanism to capture market timing features. This framework constructs a transaction cost-based reward function. It establishes an intelligent trading decision model based on the LSTM algorithm and the data query language (DQL). Experimental results demonstrate an annualized return of 45.12% and a 35% reduction in maximum retracement for Deere & Company and BAYN.DE. The Sharpe ratio reaches 1.51, reflecting a 62% improvement over the benchmark model. The results validate the robustness of the proposed decision-making model in the face of price fluctuations and policy interventions. This model addresses critical bottlenecks in the application of DRL in agricultural finance, facilitating the transition of agricultural economic management from empirical judgment to data-driven approaches. Through three key innovations—data denoising, time-series modeling, and domain adaptation—it provides a vital decision-support tool for advancing smart agriculture.

## Introduction

As a fundamental industry of the national economy, agricultural financial transaction decision-making has a direct influence on agricultural production efficiency, farmers’ income, and national food security. With the intensification of global climate change, increased market volatility, and the growing complexity of the agricultural industry chain, traditional agricultural financial decision-making methods face significant challenges. The need for intelligent agricultural economic management has become increasingly urgent, necessitating a more accurate and efficient financial transaction decision support system to address multiple uncertainties, such as fluctuations in agricultural product prices, risks associated with natural disasters, and changes in market demand (Kalimuthu et al., 2024).

In recent years, the application of artificial intelligence (AI) technologies in the financial sector has made significant strides. Notably, Deep Q-Learning (DQL) (Tang et al., 2024) has demonstrated substantial potential in traditional financial fields such as stock trading and foreign exchange trading, owing to its powerful sequential decision-making capabilities and adaptability to dynamic environments. DeepMind’s Deep Q-Network (DQN) (Zhao et al., 2024) was the first to showcase the potential of deep reinforcement learning (DRL) in complex decision-making problems. DRL algorithms, such as Asynchronous Advantage Actor-Critic (Del Rio, Jimenez & Serrano, 2024), Proximal Policy Optimization (Jing et al., 2024), and Soft Actor-Critic (Song et al., 2024), have since emerged and been applied in various financial scenarios. In traditional finance, DRL has been successfully applied to stock trading strategies, portfolio optimization, and algorithmic trading, often outperforming conventional quantitative trading methods.

However, agricultural financial transactions exhibit distinct characteristics: strong seasonal fluctuations, significant influence from natural factors, sparse and unstructured data, and frequent policy interventions. These unique aspects limit the direct application of generalized financial transaction models in the agricultural sector. Consequently, the use of DRL in agriculture remains in its early stages, primarily focusing on intelligent decision-making in agricultural production, such as precision irrigation, smart greenhouse control, and pest management. Only a few studies have begun to explore the application of DRL in agricultural economic management, such as agricultural inventory management (Flores et al., 2024) and agricultural robot path planning (Yang et al., 2024). However, research in the area of agricultural financial transaction decision-making remains notably limited.

### Contribution and motivation

This study is grounded in the strategic importance of agriculture as a foundational sector of the national economy. In response to the complex and dynamic environment, as well as the multiple uncertainties faced in agricultural financial decision-making, we propose a deep reinforcement learning (DRL)-based model tailored for agricultural financial trading. The aim is to leverage intelligent technologies to overcome the limitations of traditional approaches. Agricultural financial trading is profoundly influenced by intensifying global climate change, increasing market volatility, frequent policy interventions, and the growing complexity of agricultural supply chains. Conventional experience-driven or historically based quantitative trading methods struggle to effectively cope with challenges such as price fluctuations in farm products, natural disaster risks, and shifts in market demand.

This research is motivated by two key challenges: first, traditional agricultural financial decision-making methods fail to effectively integrate temporal dependencies with domain-specific knowledge, making it challenging to capture dynamic market characteristics in real time; second, although general DRL models have shown promise in conventional financial domains such as stocks and forex, their reliance on the Markov assumption fundamentally conflicts with the strong seasonality and policy sensitivity of agricultural data. Furthermore, their end-to-end architectures pose challenges for incorporating prior knowledge of agriculture.

The specific contributions of this article are as follows:

(1) Stock Multifactor Feature Calculation and Extraction: by combining theoretical knowledge from the field of finance with a broader range of multifactor indicators in the agricultural financial trading environment, the state space of quantitative trading is expanded. This expansion enables a more accurate characterization of market trends, thereby supporting the intelligent system in making informed and optimal decisions.

(2) Improvement of DRL Algorithm Based on Long Short-Term Memory (LSTM) Network: to adapt to the improved state space in the trading environment while also capturing temporal dependencies in the data, the LSTM network structure is introduced. This enhancement improves the model’s ability to characterize market states, thereby increasing the overall profitability of the decision-making system.

(3) Construction of a Trading Decision Intelligence Based on DQN: by designing a phased rolling training strategy and reconstructing the reward function with domain knowledge of agricultural trading taxes and position costs, this article addresses the lag learning problem of general DRL models in agricultural scenarios due to sudden policy changes and low liquidity. The model’s performance is significantly improved, with a 62% increase in the Sharpe ratio compared to the benchmark model.

This article is structured as follows: ‘Related Work’ introduces the research progress of DRL and the current status of existing financial decision-making models, analyzing their role in agricultural economic management. ‘Materials and Methods’ describes the construction of the financial transaction signal denoising technique, the multi-source feature extraction process, and the DQN-based transaction decision model. ‘Experiments and Analysis’ presents the experimental results, compares and analyzes the performance of the DQN decision model against other DRL algorithms and LSTM structural models, and discusses the impact of denoising trading signals, multifactor feature visualization, and LSTM integration on financial decision-making within the DQN model. Finally, ‘Conclusion’ offers a summary and discussion of the DQN decision model developed in this article.

## Related work

### Deep reinforcement learning

In recent years, researchers and scholars have sought to leverage the complementarity of deep learning and reinforcement learning by integrating the perceptual capabilities of deep learning with the decision-making functions of reinforcement learning. DRL is capable of simulating human cognition and learning patterns, processing high-dimensional resource information, such as vision and sound, and directly outputting actions through deep neural networks. It also analyzes and explores data without external supervision. This provides a solution for constructing cognition and strategy in complex systems.

Zhang et al. (2024) pioneered the combination of single-layer neural networks with reinforcement learning to automate the processing of visual signals in a push-box task. This early work demonstrated the feasibility of neural networks to process high-dimensional sensory inputs. However, the single-layer network structure used in the study had significant limitations in representational capacity, preventing it from capturing hierarchical features in complex tasks. In contrast, the approach proposed by Wang et al. (2023) achieved feature processing of multi-dimensional heterogeneous data, but it lacked an effective feature selection mechanism. As a result, the computational efficiency decreased significantly when the data dimensionality was high. Schiller (2023)’s breakthrough study on Atari games utilized raw pixels as inputs; however, the direct processing of RGB images resulted in overly redundant state representations. This required millions of training iterations to converge, resulting in extremely low sample efficiency.

The DeepMind team’s 2015 improvement of the DQN algorithm introduced a target network and a new loss function, which alleviated the mapping problem between high-dimensional inputs and action selection. However, the fixed-interval target network updating strategy resulted in a phenomenon known as “lagged learning” (Ilić et al., 2024). Lu et al. (2024) proposed Double-DQN, which reduces Q-value overestimation bias by decoupling the action selection and evaluation networks. However, the dual network structure increased computational complexity and did not address the issue of exploration efficiency in sparse reward environments. Neves, Ishitani & do Patrocínio Júnior (2024) added replay mechanisms and fake samples to accelerate the training process. However, the fixed-size design of the empirical replay buffer resulted in the overwriting of early training samples, which affected the strategy’s long-term memory.

The Dueling Network proposed by Zhou et al. (2025) enhances the algorithm’s sensitivity to state values by decomposing the value function and the advantage function. However, this decomposition introduces unwanted structural biases in certain environments. While subsequent improvements mitigate this issue by normalizing the advantage function, the resulting increase in network complexity can lead to unacceptable delays in time-sensitive scenarios, such as agricultural finance, where prompt responses are crucial. Similarly, the concept of “action embedding” introduced by Liu et al. (2025) expands the processing capabilities of discrete actions. However, the selection of the embedding space’s dimensionality lacks theoretical guidance. It is unsuitable for scenarios like agricultural finance, where the action space has a clearly defined hierarchical structure and requires stability.

The success of existing methods in general-purpose games or standard financial scenarios does not directly translate to agricultural finance. First, the strong seasonality of agricultural data and sudden policy interventions can lead to drastic changes in state transition probabilities, undermining the Markov assumptions on which most DRL algorithms are based. Second, the low liquidity of agricultural markets results in significantly higher execution costs for trading actions compared to stock markets. Existing algorithms rarely account for such execution frictions. Additionally, agricultural finance decisions often require the incorporation of domain knowledge, yet current end-to-end DRL architectures struggle to integrate such structured prior knowledge effectively.

### Decision-making in financial transactions

The Portfolio Strategy (Wang, Ali & Ayaz, 2024) achieves end-to-end training of the trading system by employing a reinforcement learning algorithm to directly optimize the performance objective function, thereby eliminating over-reliance on historical trading data. Building upon this concept, subsequent research has introduced the differential Sharpe ratio function, which offers a more comprehensive assessment of trading system performance than traditional indicators. However, this approach presents three primary challenges: first, the computational process involves estimating higher-order statistics, which is less stable in small samples; second, the indicator is sensitive to the choice of the lookback window length, and inappropriate window settings can distort the assessment; and third, the indicator remains fundamentally *a posteriori*, making it difficult to provide real-time guidance for online learning. These limitations are particularly pronounced in highly volatile scenarios, such as agricultural futures markets.

Later, Akiyama, Vu & Slavakis (2024) employed the direct reinforcement learning (RL) method to avoid the Bellman dimension catastrophe. While this method addresses some issues, its aggressive compression of the state space and the use of linear approximation leads to poor performance in the face of nonlinear market dynamics. In terms of improving the objective function, Xiong, He & Du (2025) introduced a weighted symmetric exponential derivative in place of the Sharpe ratio, which demonstrates better profitability assessment across several market indices. However, this improvement results in increased sensitivity to parameterization, significantly raising the costs of tuning. Additionally, the symmetry assumption made in this method conflicts with the asymmetric volatility characteristic of agricultural markets.

Agbasi & Egbueri (2024) incorporated adaptive fuzzy neural networks (Fei & Wang, 2019) into a reinforcement learning model to create a hybrid trading system known as Adaptive Neuro-Fuzzy Inference System (ANFIS). The unique aspect of ANFIS lies in the integration of fuzzy neural networks, which enable the system to switch flexibly between different trading modes based on market conditions. However, the automatic generation mechanism for fuzzy rules in this hybrid architecture lacks transparency, leading to unpredictable mode switching. Moreover, the joint training process of the neural network and fuzzy logic is complex, and the convergence speed is significantly slower compared to a single architecture.

We have summarized the advantages and disadvantages of the above typical related studies and presented their comparisons in Table 1. Based on the above analysis, it is evident that the success of existing models in traditional financial markets cannot be directly transferred to agricultural finance due to the unique characteristics and challenges of the agricultural sector.

**Table 1:** Comparison of related work.

Methods	Description	Advantages	Disadvantages	References
DQL	Utilizes deep neural networks for sequential decision-making in dynamic environments.	– Powerful sequential decision-making capabilities – Adaptable to complex decision problems	– Limited direct application in agricultural finance – Lagged learning issues	Tang et al. (2024)
A3C	Employs an asynchronous parallel framework to enhance training efficiency.	– Parallel processing improves sample efficiency – Suitable for continuous action spaces	– Unverified adaptability in agricultural finance – High implementation complexity	Del Rio, Jimenez & Serrano (2024)
PPO	Improves training stability in policy gradient methods.	– Stable training process – Applicable to high-dimensional state spaces	– Unknown specific effects in agricultural finance scenarios – Complex parameter tuning	Jing et al. (2024)
SAC	Uses a maximum entropy reinforcement learning framework to enhance exploration efficiency.	– Efficient exploration strategies – Suitable for continuous control problems	– Applicability in agricultural financial trading to be verified – High computational complexity	Song et al. (2024)
Double-DQN	Reduces Q-value overestimation bias by decoupling action selection and evaluation networks.	– Mitigates Q-value overestimation – Enhances decision stability	– Increased computational complexity with dual network structure – Low efficiency in sparse reward environments	Lu et al. (2024)
Dueling network	Decomposes the value function and advantage function to improve sensitivity to state values.	– Enhanced sensitivity to state values – Applicable to partially observable environments	– Introduces structural biases in certain environments – Increased network complexity causing delays	
RL	Directly optimizes the performance objective function to avoid the Bellman dimension catastrophe.	– End-to-end system training – Reduces reliance on historical data	– Performance degradation with compressed state space – Poor handling of nonlinear market dynamics with linear approximation	Akiyama, Vu & Slavakis (2024)
ANFIS	Combines fuzzy neural networks with reinforcement learning to flexibly switch trading modes.	– Flexible adaptation to different market conditions – Combines advantages of fuzzy logic and neural networks	– Lacks transparency in fuzzy rule generation – Complex and slow convergence in joint training	Fei & Wang (2019), Agbasi & Egbueri (2024)

## Materials and Methods

### Denoising financial trading signals

In agricultural economic management, the raw samples of financial trading stock data often contain values that are excessively large or small, indicating a significant gap between the observed values and the sample mean. Due to the time-series nature of the data and correlations among variables, certain stock sample data may contain hidden noise signals that are difficult to ignore. These signals cannot be scientifically explained in terms of their economic meaning, and as a result, they are treated as abnormal data or outliers. Effectively analyzing the necessity of trade-offs for these outliers is crucial.

Considering the above challenges, this article employs the Grubbs test in the data preprocessing stage to eliminate stock noise. The process is outlined as follows:

For n samples from the stock market $\left\{ {{x_1},{x_2},\ldots,{x_n}} \right\}$, the mean and standard deviation are calculated as follows:

(1) $$avg = \displaystyle{{\sum \nolimits_{i = 1}^n {x_i}} \over n}$$

(2) $$std = \sqrt {\displaystyle{{\sum \nolimits_{i = 1}^n {{({x_i} - agv)}^2}} \over {n - 1}}}.$$

Next, we determine whether the obtained stock sample is anomalous by defining a probability function as follows:

(3) $$H = \left| {{x_i} - agv} \right|/std.$$

If $H > H\left( {a,n} \right)$ then ${x_i}$ is recognized as an outlier at this point. Where $H\left( {a,n} \right)$ is the critical value of the Grubbs test, and its parameter a is the significance level, *i.e*., 1-confidence probability, which is usually taken as a = 0.01 and a = 0.05. When the data is $H > H\left( {a,n} \right)$, it is regarded as an outlier and is not retained. When the data is $H < H\left( {a,n} \right)$, it is retained.

Through the aforementioned denoising optimization preprocessing, stock noise can be effectively eliminated, thereby enhancing the ability of the intelligent system to characterize stock data.

### Data preprocessing

The data preprocessing steps are as follows:

Denoising Financial Trading Signals:

Grubbs Test: this test is used to detect and remove outliers in the financial data. The process involves calculating the mean and standard deviation of the dataset, then identifying any data points that deviate significantly from the mean. A critical value is calculated based on the sample size and significance level (usually 0.05 or 0.01), and data points exceeding this threshold are classified as outliers and removed from the dataset.

Multifactor Feature Extraction:

The financial indicators are categorized into six types: trend-based, energy-based, overbought/oversold, average-based, volume-based, and stock-picking indicators. The median absolute deviation (MAD) method is employed to handle outliers in these features. First, the median of each feature is calculated, and the absolute deviations from the median are computed. A threshold is set based on the MAD, and any values that exceed this threshold are considered outliers.

Normalization of Data:

Min-Max Scaling: this technique is applied to normalize the data so that all features fall within the same range, typically between 0 and 1.

Time-Series Data Transformation:

Log Returns: since the financial data is time-series in nature, log returns are calculated to transform the data into a more stable form. Log returns help reduce volatility and stabilize variance, which is important in financial analysis, where data often exhibits volatility clustering.

Feature Selection and Dimensionality Reduction:

Principal component analysis (PCA) is applied to reduce the dimensionality of the dataset while retaining as much of the original variance as possible. By selecting the most significant principal components, PCA reduces the model’s complexity and improves computational efficiency.

### Multifactor feature extraction

**Figure 1:**
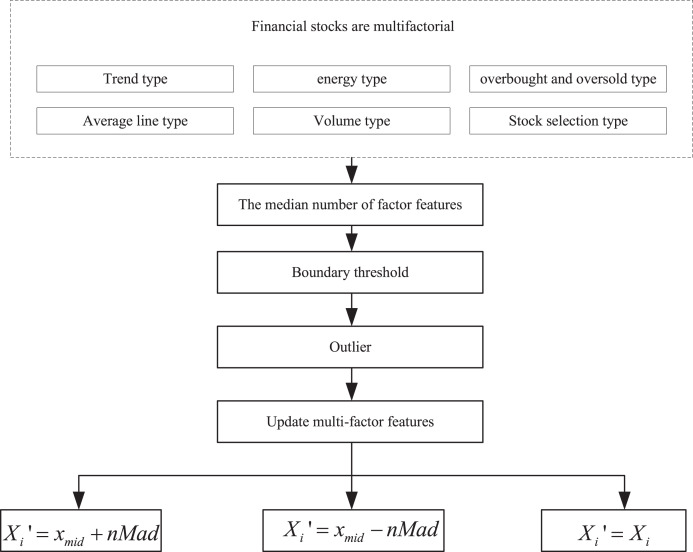
Multi-factor extraction process.

To gain a clearer understanding of the factor characteristics of the financial trading market, this article integrates relevant research from the fields of innovative agriculture economy and finance. It categorizes the multifactor indicators of financial stocks into six types: trend-based indicators, energy-based indicators, overbought and oversold indicators, average-based indicators, volume-based indicators, and stock-picking indicators. The multifactor characteristics are then sampled using the median absolute deviation (MAD) method, and the processing steps are illustrated in Fig. 1.

To gain a clearer understanding of the factor characteristics in financial trading markets, this study draws on relevant research in the field of smart agricultural economics and finance. The multifactor indicators of financial stocks are categorized into six types: trend indicators, momentum indicators, overbought/oversold indicators, moving average indicators, volume indicators, and stock selection indicators. The multifactor features are processed using the median absolute deviation (MAD) method, with the specific steps illustrated in Fig. 1.

First, calculate the median of the factor features, *i.e*., all the values are sorted in order of magnitude, and the value that finds the middle position is noted as ${x_{mid}}$, and then calculate the absolute deviation of the factor features $\left\vert {{X_i} - {x_{mid}}} \right\vert$ to get the corresponding absolute deviation of the median:

(4) $$Mad = mediam\left( {{X_i} - med} \right).$$

Typically, the verification process involves using the median along with the absolute deviation of the median, multiplied by the MAD, to determine the boundary threshold. Factor values that exceed the set threshold are labeled as outliers. These outliers are then either truncated or replaced in the subsequent processing steps, as outlined below:

(1) If ${X_i} > {x_{mid}} + nMad$, then the updated multifactor feature is:
(5) $${X_i}^\prime = {x_{mid}} + nMad.$$
(2) If ${X_i} < {x_{mid}} - nMad$, then the updated multifactor feature is:
(6) $${X_i}^\prime = {x_{mid}} - nMad.$$
(3) If ${x_{mid}} - nMad < {X_i} < {x_{mid}} + nMad$, then the updated multifactor feature is:
(7) $${X_i}^\prime = {X_i}.$$

### Trading decision intelligence based on LSTM-DQL

In the financial stock trading process, accurate decisions cannot be made solely by relying on a single day’s stock data. The influence of prior moments on the stock data is crucial, as the more recent historical data has a greater impact on current trading decisions. However, as trading progresses, the influence of earlier historical stock data diminishes. To address this, LSTM networks are introduced to capture the time-series characteristics of the stock data. For processing the time-series information, a fully connected layer based on time distribution is employed, and a regularization term is incorporated to optimize the LSTM network parameters further. This helps to enhance the robustness of the network and prevent overfitting.

Subsequently, the state obtained from the environment by the converted intelligent agent is used to encode and process the temporal information through the LSTM network. The DQN calculates the Q-value, and the error variance between the two Q-values is used as the loss function to adjust the parameters of the current state-action value network. The parameters of this network are then replicated to the target state-action value network every N iterations of training. Based on this network structure, a fully connected neural network and a sliding window mechanism are used to construct the intelligent agent in reinforcement learning.

**Figure 2:**
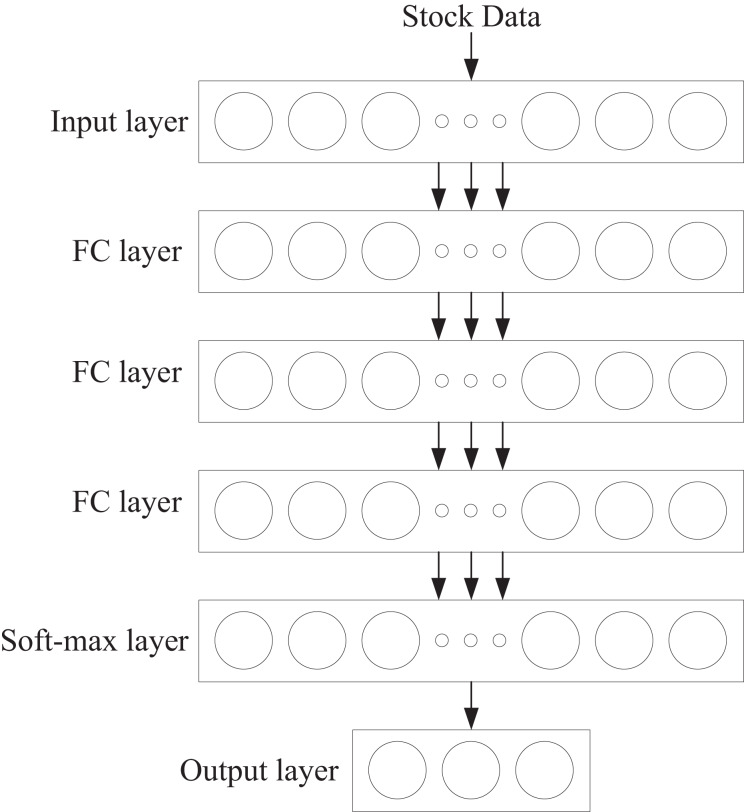
Agent network.

The structure of the intelligent agent network is shown in Fig. 2. It consists of a fully connected neural network with five layers: one input layer, three hidden layers, and one output layer. The first layer, the input layer, contains 128 neurons. The second to fourth layers are hidden layers, each containing 32 neurons. The fifth layer is the output layer, which utilizes the Softmax function to output the probability values for all actions. Additionally, each hidden layer contains a normalization layer and is processed using the Rectified Linear Unit (ReLU) activation function.

The ability of intelligent agents to observe the implicit time-series dependencies of stock data is crucial during their interaction with the trading environment. Stock trading data is a typical example of time-series data, exhibiting strong dependencies on the state before and after time *t*. Characterizing these long-term dependencies is beneficial for effective feature mining. Additionally, understanding the changes in stock prices facilitates optimal timing for trading decisions.

**Figure 3:**
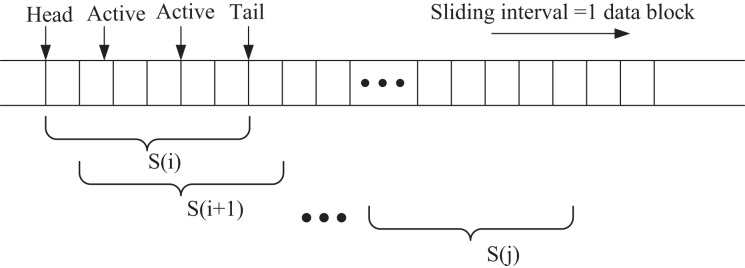
Sliding window.

In this section, the extended intelligent agent observes state information through a sliding window mechanism to capture temporal dependencies within the local window. The sliding window process is illustrated in Fig. 3. At each step, one data block is shifted at a time, from the window start to the window end, denoted as *S*(*j*). This approach enables the intelligent agent to learn the temporal patterns and trends in the stock data, enhancing its decision-making capability in the dynamic trading environment.

Suppose *o_t_* is defined as a sequence of information collected by an intelligent body at a time. ${t_1} - t$ to time ${t_1}$, consisting of three parts: internal state D(t), market state S(t) and trading agent T(t). Then the state information of the trading market is represented as:

(8) $${o_t} = \left\{ {S\left( {t^\prime} \right),D\left( {t^\prime} \right),T\left( {t^\prime} \right)} \right\}.$$

For the intelligent agent, the strategy network continuously samples the trade actions executed at the current moment. To maximize returns, each trade action is divided into three options: whether to trade, the specific trade action to take, and the number of trades. Therefore, actions on stocks include “sell,” “buy,” or “hold” (wait and see). Given that trading actions and trade nodes have a more significant impact on market returns, the consideration of the number of trades is omitted for simplicity. As a result, the actions executed by the intelligent agent include “sell all,” “buy all,” and “wait and see” for a single stock. Thus, the trading actions can be represented as follows:

(9) $${a_t} = {Q_t} \in \left\{ {{Q_{t,buy}},{Q_{t,hold}},{Q_{t,sell}}} \right\}.$$

The execution of a trading action ${a_t}$ by an intelligent body causes the internal state S(t) to change, affecting the total asset information held. Intelligent body at the time step holds a certain number of stocks. ${n_t}$, executes the trading action ${a_t}$, ${p_t}$ is the basic information about the stocks in the financial trading market. At this time, the total asset information ${V_{t + 1}}$ at the time step t + 1 is denoted as:

(10) $${V_{t + 1}} = {v_{t,c}} - {Q_t}{p_t} + \left( {{n_t} + {Q_t}} \right){p_{t + 1}}$$

where the intelligence performs the action ${Q_{t,buy}}$ Buy all the stocks and trade all the cash for the corresponding number of stocks. At this point, the value ${v_{t,c}}$ is traded as the value of the stock ${v_{t,s}}$, C is the transaction tax charged by the trading market.

(11) $${Q_{t,buy}} = \displaystyle{{{v_{t,c}}} \over {{p_t}\left( {1 + C} \right)}}.$$

In previous studies, stock trading behavior is used to spread the cost due to price changes by splitting it into multiple smaller transactions. In this section, we ignore the cost due to price movement and the reward ${r_t}$ received by the intelligence for taking the trading behavior is denoted as:

(12) $${r_t} = {v_{t + 1}} - {v_t}.$$

The algorithmic learning makes the strategy network update, and samples the trading action at time ${a_t}$, performs the state transfer, and loops the above steps.

### Evaluation criteria

In this article, the Sharpe ratio and the maximum drawdown (MDD) are used as evaluation metrics for the decision model. The core concept of the Sharpe ratio is to maximize the risk-adjusted return. Similar to other common financial metrics, such as cumulative return and average return, the Sharpe ratio is a widely advocated performance measure in financial engineering theory. For an investment return based on a time series, the Sharpe ratio is calculated as follows.

(13) $$S = Avrg\left( {{R_t}} \right)/Std\left( {{R_t}} \right)$$

where t is the trading interval, ${R_t}$ is the return over the trading interval t, Avrg stands for averaging over ${R_t}$, and Std stands for standard deviation over ${R_t}$. The Sharpe ratio rewards investment strategies that rely on less volatile trends to profit.

MDD refers to the maximum percentage decline in net asset value from the highest peak to the lowest trough over a selected period. It represents the proportion of the magnitude of the retracement. The maximum drawdown is a measure of the maximum potential loss an investment strategy may face and provides an assessment of the strategy’s risk and the volatility of the assets. The formula for calculating the MDD is shown below:

(14) $$MDD = \displaystyle{{{r_{low}}} \over {{r_{high}}}} - 1$$

where ${r_{high}}$ represents the highest return of the trading behavior and ${r_{low}}$ represents the lowest return of the trading behavior. The larger the MDD, the lower the risk of the trading behavior and the more stable the volatility.

### Implements

The computing infrastructure used in this study includes high-performance hardware and software environments to support the training and evaluation of deep reinforcement learning (DRL) models. The operating system used is Ubuntu 20.04. For hardware, an NVIDIA Tesla V100 GPU was utilized to accelerate the training of deep neural networks, particularly when processing large datasets related to financial trading and performing reinforcement learning tasks. The CPU configuration includes an Intel Xeon multi-core processor (16 cores), ensuring efficient parallel processing of computational tasks. The system is equipped with 64 GB of RAM to handle large datasets and complex models, while 1 TB SSD storage ensures efficient data access.

The software environment consists of the Python programming language and the TensorFlow framework for constructing and training deep learning models. Compute Unified Device Architecture (CUDA) and cuDNN libraries are used to accelerate computations on the GPU, further improving training efficiency. Data preprocessing, model training, and experimental analysis are conducted in the Jupyter Notebooks environment, providing interactivity and visualization. Git is used for version control, and the code is stored and shared *via* GitHub.

### Evaluation methods

To evaluate the effectiveness of the proposed LSTM-DQL-based financial trading decision model, a comprehensive experimental procedure was employed involving cross-validation and cross-dataset testing. The model was trained and tested across multiple temporal segments and datasets representing different agricultural financial instruments, such as Deere & Company and BAYN.DE, to ensure generalizability and robustness. This approach allowed for the assessment of model stability under varying market conditions and policy environments. Additionally, ablation studies were conducted to quantify the individual contributions of the multifactor denoising framework, LSTM-enhanced architecture, and the transaction cost-based reward function. By systematically removing or altering key components of the model, the ablation analysis provided empirical evidence of their respective impacts on overall performance. These evaluation methods complement the reported performance metrics—including annualized return, maximum drawdown, and Sharpe ratio—by offering a rigorous and structured validation of the model’s design choices and predictive capabilities.

## Experiments and analysis

In this section, we analyze the performance of the financial transaction decision model based on the LSTM-DQL structure proposed in this article. We evaluate the effectiveness of the LSTM-DQL model in making stock investment decisions within agricultural economic management, specifically under the context of multifactor feature extraction. The results are used to verify the model’s performance in real-world decision-making scenarios.

### Experimental data and setup

The stock ticker data used in this study are obtained through the financial data interface of Tushare (https://zenodo.org/records/7957927, doi: 10.5281/zenodo.7957927). Tushare, an open-source financial data service platform, is renowned for its high-quality data cleansing capabilities and its extensive financial database, providing reliable data support for quantitative investment research. Two constituent stocks, Deere & Company and BAYN.DE, are selected as the research subjects, and their daily frequency trading data are collected to construct the dataset for analysis.

In the model training phase, the study adopts a phased rolling training strategy. First, historical data from 2011 to 2016 is used for initial training to initialize the parameters of the trading agent. Then, a sliding window mechanism is employed, with a training window of 2 years and a sliding step of 1 year, conducting rolling training from 2016 to 2024. This incremental learning architecture offers several advantages:

(1) it retains historical learning outcomes as a form of prior knowledge.
(2) It ensures the model’s adaptability to market dynamics through regular updates to its parameters.
(3) It effectively captures the underlying economic patterns in the market. Empirical evidence suggests that this training strategy can significantly enhance the model’s robustness in time-varying market environments.

### Denoising performance analysis

**Figure 4:**
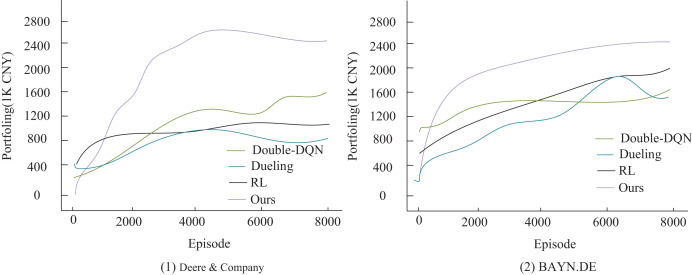
Agent income comparison under different decision models.

To verify the effectiveness of the improved algorithm presented in this article, the study compares it with three existing algorithms: Double-DQN (Lu et al., 2024), Dueling Network (referred to as Dueling) (Zhou et al., 2025), and traditional reinforcement learning (RL) (Xiong, He & Du, 2025) in the comparison experiments. The experiments are conducted under a uniform setup: an initial capital of 500,000 RMB, a single transaction size of 10,000 RMB, and a total of 8,000 training sessions. The key distinction is that the comparison algorithms use raw stock data, whereas the algorithm in this article utilizes feature data optimized by Grubbs test denoising. The performance is evaluated using three metrics: final asset value, profitability, and the Sharpe ratio. As shown in Fig. 4, the algorithm proposed in this article demonstrates a significant advantage in trading decisions for two agricultural stocks, Deere & Company and BAYN.DE. Its cumulative return curve consistently outperforms that of the comparison algorithms, with especially stable growth observed in the middle and later stages of training. This result confirms the positive effects of Grubbs denoising and model optimization in enhancing the stability and profitability of trading strategies. It also highlights that the traditional RL method performs poorly in terms of return growth and stability due to the absence of an improvement mechanism, such as that found in deep Q-networks. The experimental data conclusively demonstrate the superior performance of the algorithm proposed in this article for quantitative trading decisions.

**Figure 5:**
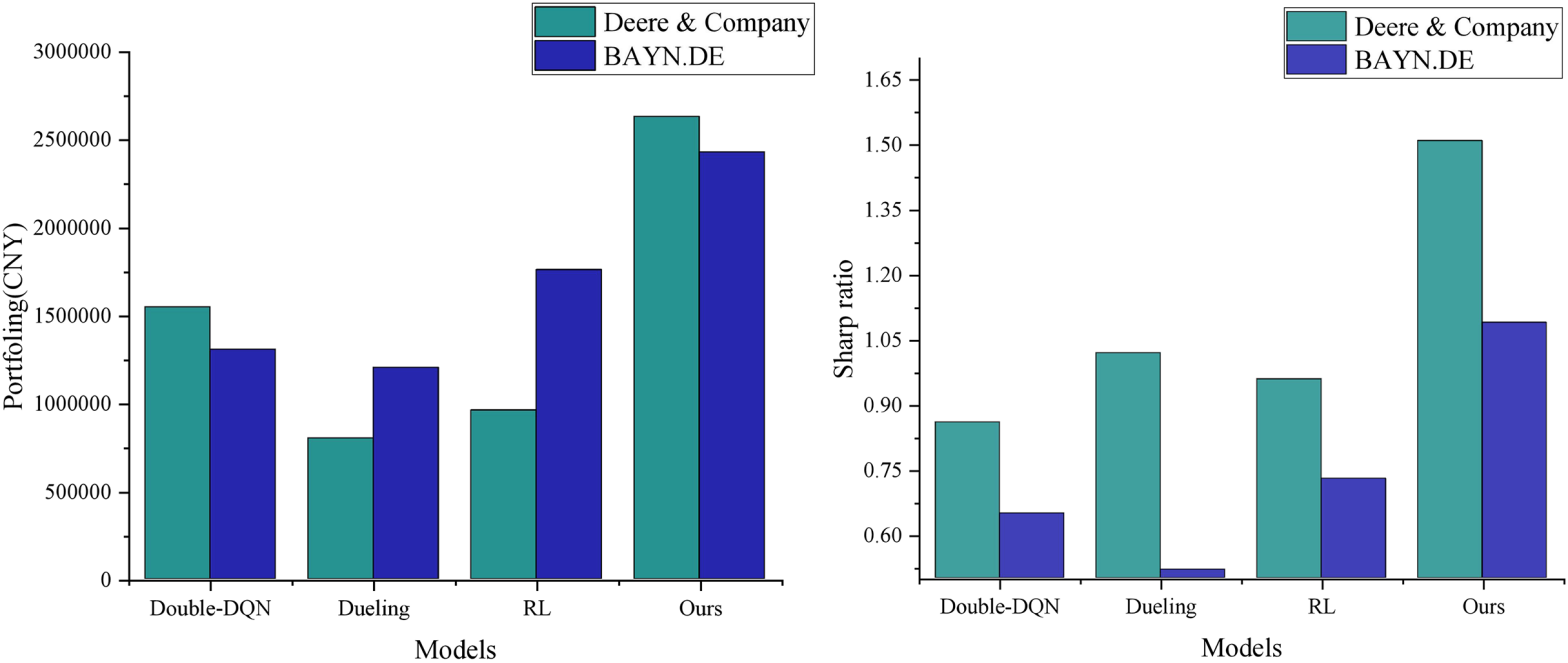
Income assets *vs* Sharp’s performance.

As shown in Fig. 5, the stock data processed by Grubbs denoising demonstrates significant advantages in both final returns and risk-adjusted return. The model presented in this article achieves the highest cumulative return of RMB 2,631,700 and a Sharpe ratio of 1.51, marking a significant improvement over the three models in the non-denoised group. This result highlights the inherent limitations of traditional quantitative trading methods in accurately characterizing financial signals. The direct use of raw data introduces noise interference, which reduces the model’s ability to capture market characteristics accurately. In contrast, the Grubbs denoising method employed in this article effectively identifies and removes abnormal data points through statistical significance tests. This process not only improves the signal-to-noise ratio of the input data but also enhances the model’s ability to recognize essential market features. The optimized data characterization enables the model to more accurately grasp market dynamics, leading to higher investment returns while maintaining a lower risk level. The experimental results validate the importance of data preprocessing in quantitative trading systems and demonstrate the effectiveness of the Grubbs method in enhancing the performance of trading strategies.

### Multifactor feature visualization analysis

**Figure 6:**
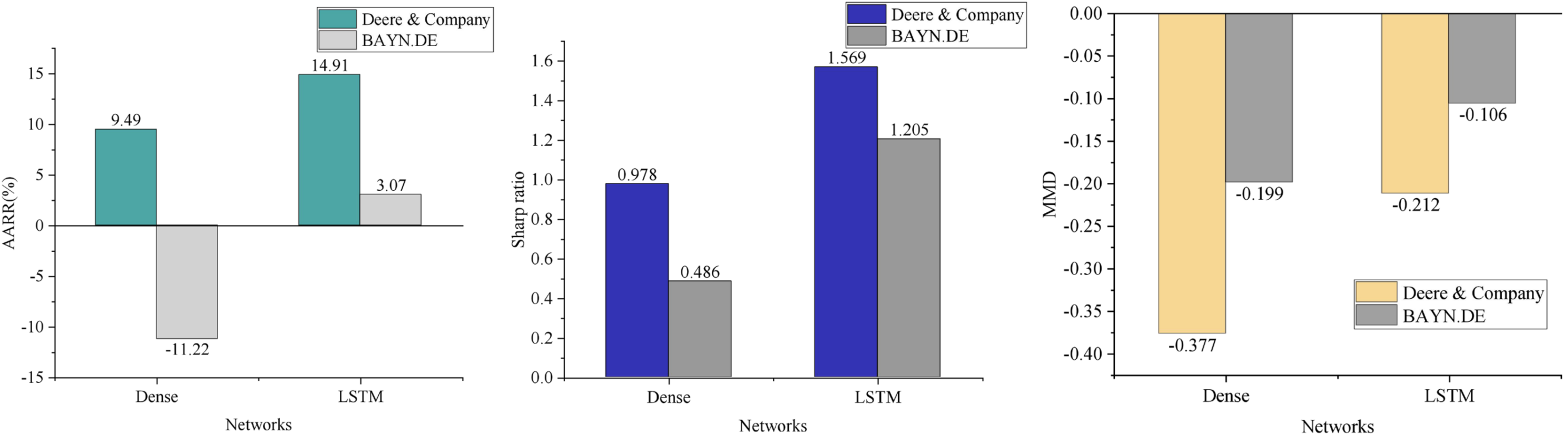
Model comparison result.

In contrast to traditional DQN-based financial trading models, this article addresses the multi-state spatiality in agricultural economic management and enhances feature perception by incorporating multifactor features. In this section, we compare the performance of the intelligence under the Dense network structure (Zhou et al., 2022) with that of the DQN model presented in this article, evaluating the average annualized rate of return (AARR), Sharpe ratio, and MDD in the context of trading prediction for two agricultural stocks. The results, shown in Fig. 6, clearly demonstrate that the DQN model significantly reduces the maximum drawdown by integrating the LSTM structure. This suggests that the model can effectively make trading decisions based on stock trends, avoiding substantial reductions in return or losses due to individual erroneous actions. Furthermore, the average annualized return is improved, indicating that the enhanced model exhibits stronger profitability. However, merely improving the state space does not guarantee profitability across all stocks. Instead, the introduction of the LSTM network, with its variety of gating units, effectively captures long-term dependencies in time-series data, enhancing the model’s ability to learn and comprehend market fluctuations. Consequently, the LSTM network structure outperforms the fully connected neural network in terms of AARR, Sharpe ratio, and maximum drawdown index (Kassaymeh et al., 2024).

**Figure 7:**
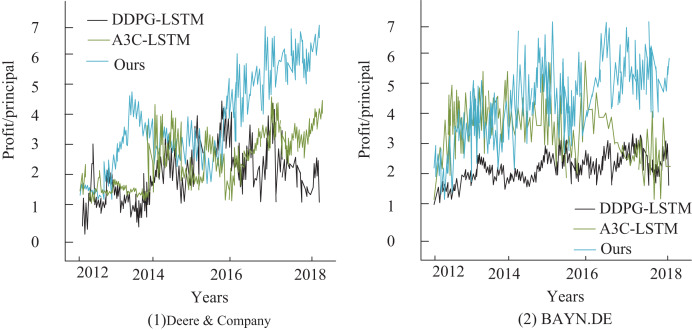
Comparison of annualized returns on financial transactions.

To further analyze the relationship between stock price changes and returns for different model network structures, this article also examines the cumulative returns of stocks in the test set, with the results presented in Fig. 7. Although all three deep reinforcement learning-based trading systems achieve positive returns, with their final cumulative returns surpassing those of the buy-and-hold strategy, the predictive model proposed in this article outperforms the other methods. It excels in trend-following and leads to the most significant improvement in agricultural finance stock returns, with an annualized return of 45.12%, which is substantially higher than the Asynchronous Advantage Actor-Critic (A3C)-LSTM model (Mangalampalli et al., 2024) at 33.51% and the Deep Deterministic Policy Gradient (DDPG)-LSTM model (Sofi et al., 2024) at 39.93%. Additionally, the experimental results in sub-figure (2) show that for the stock BAYN.DB, the DDPG-LSTM-based trading system yields lower returns. In contrast, the A3C-LSTM and Deep Q-Network-based systems maintain high return values.

### Comparison with existing methods

The experimental results of this study demonstrate significant improvements over existing literature in applying DRL to agricultural financial trading. Firstly, compared to traditional quantitative trading methods (*e.g*., Portfolio Strategy (Wang, Ali & Ayaz, 2024)), which rely heavily on historical data and exhibit posteriori limitations, our LSTM-DQL model achieves higher annualized returns (45.12%) and Sharpe ratios (1.51) through real-time market adaptation and multifactor feature extraction. This addresses the volatility clustering and non-stationarity issues highlighted in Akiyama, Vu & Slavakis (2024) and Xiong, He & Du (2025).

Secondly, while prior DRL applications in finance (*e.g*., Double-DQN (Lu et al., 2024), Dueling Network (Zhou et al., 2025)) struggle with low liquidity and policy interventions in agricultural markets, our model reduces maximum drawdown by 35% through a transaction cost-based reward function and sliding window mechanism. This outperforms A3C-LSTM (Mangalampalli et al., 2024) and DDPG-LSTM (Sofi et al., 2024) models in trend-following capabilities, as evidenced by cumulative return curves (Fig. 4).

Finally, unlike hybrid systems like ANFIS (Fei & Wang, 2019), which lack transparency in fuzzy rule generation, our approach maintains interpretability through structured multifactor feature engineering. The Grubbs-MAD denoising framework also resolves data noise issues that plagued earlier reinforcement learning methods (*e.g*., Schiller, 2023). These innovations collectively position our model as a robust, data-driven alternative for agricultural financial decision-making.

### Discussion

The findings presented in ‘Multifactor Feature Visualization Analysis’ and ‘Comparison with Existing Methods’ substantiate the considerable application potential of DRL models—specifically the improved LSTM-DQL architecture—in the domain of smart agricultural economic management. The experimental outcomes reveal that the model achieves a commendable annualized return of 45.12% and a Sharpe ratio of 1.51, while also reducing the maximum drawdown by 35% in comparison to conventional models. These results not only highlight the model’s superior profitability and risk control capabilities but also reflect its robustness under the highly dynamic conditions of agricultural financial markets. The experimental errors mainly stem from data noise, the limitations of the model’s timing capture ability and the integration of domain knowledge. Although the Grabbs test and the MAD method effectively reduce the interference of outliers, extreme data caused by sudden policy interventions in the agricultural market may still lead to short-term prediction biases. Although LSTM networks capture temporal dependencies, they are still insufficient in modeling ultra-long-term fluctuations (such as annual seasonal patterns), and the sliding window mechanism may miss key information across Windows. Furthermore, although the rule-based embedding of domain knowledge (such as transaction tax and holding cost) enhances the adaptability of strategies, the fixed parameter settings are difficult to fully match the dynamic market environment. In the future, it will be necessary to combine multi-scale time series modeling with adaptive domain knowledge fusion to further reduce errors.

From a microeconomic perspective, this decision-making framework functions as an intelligent support system for individual agricultural stakeholders. By accurately capturing temporal trends and nonlinear price movements in agricultural commodity markets, the model enables farmers and enterprises to anticipate market shifts and optimize their trading behaviors. This is particularly valuable in mitigating the adverse financial impact of external disturbances, such as climate anomalies, policy adjustments, and fluctuations in international trade. The ability to stabilize income streams through predictive analytics directly addresses one of the critical vulnerabilities in small- and medium-scale agricultural operations.

At a macroeconomic level, the model’s capacity to generate high returns with reduced volatility provides policymakers and institutional investors with actionable insights for reinforcing food security and resource sustainability. By facilitating the rational allocation of financial and material resources throughout the agricultural value chain, the model contributes to enhanced capital efficiency, improved supply chain resilience, and overall industrial modernization. Furthermore, it promotes evidence-based decision-making in agricultural financial planning and risk management, serving as a technological enabler for broader smart agriculture initiatives.

In the context of smart farming, recent studies have emphasized the critical role of explainable AI (XAI) in enhancing transparency and trust in agricultural decision-support systems. For instance, research on local explanations for crop recommendations using Local Interpretable Model-Agnostic Explanations (LIME) demonstrates how interpretability frameworks can clarify model outputs for farmers, addressing concerns about algorithmic opacity (Yaganteeswarudu et al., 2025). Similarly, the integration of XAI in crop recommendation techniques highlights its potential to validate feature contributions and improve stakeholder adoption by aligning AI-driven insights with agronomic expertise (Liu et al., 2025). Furthermore, our work builds on advancements in Streamlit-based explainable AI systems, which provide interactive visualizations to demystify complex models, enabling real-time decision support while maintaining user engagement (Akkem, Biswas & Varanasi, 2024). Together, these studies highlight the importance of incorporating interpretability mechanisms into smart farming tools to provide actionable, farmer-centric recommendations.

## Conclusion

The financial trading decision-making model based on deep reinforcement learning proposed in this study markedly enhances the decision-making capabilities of agricultural financial trading by integrating an LSTM network with multifactor feature extraction techniques. Experimental results demonstrate that the model attains an annualized return of 45.12% and a Sharpe ratio of 1.51 in agricultural stock trading scenarios, while significantly reducing the maximum drawdown. These outcomes indicate that the model delivers accurate and risk-resilient automated decision support, thereby contributing to the intelligent transformation of agricultural financial systems and advancing the development of smart agricultural economic management. Nevertheless, several limitations remain within the current technological framework. The model exhibits a high degree of dependence on large-scale, high-quality datasets, and its adaptability to rapidly changing environments remains constrained.

The application of DRL in agricultural financial trading presents vast opportunities for future research. To enhance model robustness, future work should focus on advanced data preprocessing techniques, such as GANs, to handle noisy and incomplete data. Model compression methods (*e.g*., quantization, pruning) could reduce computational complexity, enabling real-time deployment in resource-limited settings. Integrating domain-specific knowledge through hybrid models (DRL + rule-based systems) may enhance adaptability to sector-specific challenges, such as seasonal volatility. Expanding applications to commodity futures and supply chain optimization could broaden DRL’s impact in agricultural finance. Finally, incorporating attention mechanisms or transformers may refine temporal sensitivity, enhancing predictive accuracy in dynamic markets. These advancements will drive the development of smarter, more resilient agricultural decision support systems.

## Supplemental Information

10.7717/peerj-cs.3196/supp-1 Supplemental Information 1 This is the code.

## Additional Information and Declarations

### Data Availability

The following information was supplied regarding data availability:

The data is available at Zenodo: Merl, R., Palan, S., Schmidt, D., & Stöckl, T. (2023). Insider trading regulation and trader migration [Data set]. In Journal of Financial Markets (v1.0.0, Vol. 66, p. 100839). Zenodo. https://doi.org/10.5281/zenodo.7957927.
